# The Relationship between Stress and Academic Self-Efficacy among Students at Elite Colleges: A Longitudinal Analysis

**DOI:** 10.3390/bs14070537

**Published:** 2024-06-26

**Authors:** Xinqiao Liu, Chen Zhu, Zifei Dong, Yunfeng Luo

**Affiliations:** 1 School of Education, Tianjin University, Tianjin 300350, China; 2 College of Art and Science, The University of North Carolina at Chapel Hill, Chapel Hill, NC 27599, USA; 3 School of Public Administration, University of Electronic Science and Technology of China, Chengdu 611731, China

**Keywords:** stress, academic self-efficacy, cross-lagged model, elite college students

## Abstract

Stress and academic self-efficacy are crucial factors in the psychological health of college students. Previous research has shown that stress is associated with academic self-efficacy, but their longitudinal relationships among students at elite colleges remain unclear. This study aimed to explore the longitudinal relationships between stress and academic self-efficacy among students from five elite colleges in China. Descriptive statistics indicated that students experienced a slight reduction in stress accompanied by a marginal increase in academic self-efficacy from the junior year to the senior year. Correlation analysis revealed that stress was negatively correlated with academic self-efficacy. According to cross-lagged models, heightened stress significantly predicted lower levels of academic self-efficacy. However, greater academic self-efficacy did not significantly predict lower stress. In conclusion, stress exhibited a unidirectional negative prediction on academic self-efficacy over time among students at elite colleges. The results of this study suggested that elite colleges should pay more attention to the mental health of students and provide appropriate guidance, such as establishing a positive mental health atmosphere in the educational environment and employing advanced technological means.

## 1. Introduction

The higher prevalence of stress among students has raised growing concerns in the academic literature [1,2]. The college period is a critical stage for students’ transition to adulthood. College students who are confronted by environmental challenges and personal responsibilities are susceptible to stress [3]. They may confront financial strain, academic stress and social conflicts [4]. The American College Health Association (ACHA) conducted health assessments of college students nationwide and reported that the proportion of students under pressure increased from 31.9% in 2018 to 34.2% in 2019, with a high proportion of 40.3% in 2020 [5]. A survey of 2831 college students from Germany and Luxembourg showed that 45% of students had elevated stress [6]. Due to COVID-19, the bleak job market has put more pressure on college students [7]. Among 3764 French university students under the COVID-19 lockdown, 22% of them experienced high perceived stress [8]. In addition, the stress of college students is not stable and fluctuates during four years of college. For instance, among 582 first-year college students, 69.07% experienced middle-decreasing stress and 15.29% experienced high-decreasing stress eight months after enrollment [9]. First-year students and senior-year students are more stressed than junior-year students [10]. According to a survey of 2473 Chinese college students, 2.91% of them experienced increased stress, while the remainder had decreased stress from the junior year to the senior year [11].

Elite colleges generally refer to colleges with outstanding academic achievements, top rankings in “world-class” education and therefore receive government policy support and financial assistance such as Harvard University and Stanford University [12]. Similarly, China has also proposed the 985 Project, which includes 39 elite colleges. Among them are Tsinghua University, Peking University, Renmin University of China, et cetera. Students from elite universities have advantages in educational achievement, career status and entrepreneurial spirit, which all indicate an increase in their future income [13,14]. However, compared to non-elite college students, students at elite colleges may be under greater pressure because many elite colleges emphasize excellence and set high performance standards for students [15]. For example, a study of suicide in Oxford University students reported that two-thirds of those students were disturbed by academic stress [16]. A mental health report revealed academic stress and perceived stress among elite college students from South Africa [17]. Chinese college students have to face fierce competition and high expectations from parents and teachers, which can increase their stress [18,19]. Although moderate stress promotes students’ learning, excessive stress is harmful to their physical and mental health as well as academic performance [20]. For example, increasing stress on students leads to overweight, sleep disorders and alcoholism [21,22,23]. In addition, stress influences students’ social relationships [11]. When dealing with stress, some students choose to bear it alone and are unwilling to share their troubles with others, which may lead to loneliness and helplessness. Some students may become irritable and anxious due to stress, which can affect their relationships with classmates and friends and even lead to social conflicts [24]. In terms of academic performance, high levels of stress result in academic fatigue and procrastination [25,26]. Conversely, students with less stress are more likely to succeed [27]. Considering the special circumstances of stress in elite college students, exploring the influencing factors of stress among students at elite colleges is urgent and necessary.

Academic self-efficacy, the key to academic success, is one of the important factors affecting students’ stress [28,29]. Self-efficacy refers to one’s speculation about their own ability to organize thoughts, emotions and actions to produce expected results [30]. Academic self-efficacy, in particular, is the subjective estimation of one’s ability to successfully attain academic goals [31,32]. It is related to students’ confidence in specific academic tasks and their sense of control over the learning process and coping strategies when facing academic challenges. Students with high academic self-efficacy tend to set more challenging goals and invest more effort in studies [33]. Moreover, these students exhibit stronger motivation and perseverance when facing difficulties and are more likely to adopt positive coping strategies, such as seeking help and adjusting learning methods rather than giving up easily [28,34]. In this way, their psychological states tend to be stable and positive with high levels of academic self-efficacy. Conversely, students with low academic self-efficacy are more likely to experience academic procrastination, and their career decision-making level is lower [35,36]. In addition, academic self-efficacy varies during the four years of college. Previous research found that the academic self-efficacy of Chinese college students decreased from the freshman year to the junior year but increased slightly in the senior year [37]. However, there is little research on the longitudinal relationship between academic self-efficacy and stress among elite college students.

### 1.1. Literature Review

The stress and academic self-efficacy of elite college students should be noted. High standards at elite colleges have increased students’ levels of stress and brought other mental health issues, such as depression and anxiety [38]. Many elite college students are gifted. Compared to typically developing individuals, these students show their talents in terms of academic performance, emotional intelligence and social skills. However, gifted students may have higher risks of mental disorders [39]. For instance, elite athletes at colleges are under considerable pressure because they must undertake training and competition tasks while pursuing full-time university studies [40]. In addition, elite college students may have greater academic self-efficacy because they often have stronger self-motivation and self-management abilities [39]. They are more willing to accept new challenges, learn from failures and optimize their learning methods. However, their academic self-efficacy may be challenged because they have higher expectations of themselves, which makes them feel more anxious and frustrated when facing academic setbacks.

Previous research has revealed a negative correlation between academic self-efficacy and stress [29]. On the one hand, when students confront pressure from various aspects such as academics and social interactions, their academic self-efficacy may be challenged [41]. Exposure to stress induces self-doubt and uncertainty regarding students’ capabilities [42]. A study of 355 older-adult college students revealed that self-efficacy promotes the ability to handle stress; therefore, students with high self-efficacy tend to experience lower levels of stress [4]. Academic stress, as a form of stress, is closely intertwined with academic self-efficacy, offering a significant perspective for exploring the relationship between stress and academic self-efficacy [43]. Previous research has indicated a significant negative correlation between academic self-efficacy and academic stress in primary and secondary school students [43,44,45]. College students with lower academic stress also tend to show higher levels of academic self-efficacy [46]. On the other hand, high levels of academic self-efficacy allow students to embrace challenges and sustain their interest in studies [47,48]. This confidence in their abilities enhances their sense of academic achievement and contributes to the cultivation of positive learning motivation [49,50,51]. They tend to perceive academic tasks as challenging but not stressful [52]. Similarly, when students have greater academic self-efficacy, academic pressure is significantly weaker [53]. Academic self-efficacy focuses on students’ self-evaluation of their academic abilities, while general self-efficacy reflects an individual’s confidence in coping with various problems. Previous studies have shown a significant negative correlation between general self-efficacy and stress, providing evidence for our research on the relationship between academic self-efficacy and stress among elite college students [54].

Longitudinal studies have demonstrated a predictive relationship between stress and academic self-efficacy [55]. The directionality of the impact between stress and academic self-efficacy can be divided into two categories based on existing research. First, there is a unidirectional prediction of stress on academic self-efficacy [56]. Social cognitive theory enumerates four factors influencing self-efficacy: practical experiences, vicarious experiences, verbal persuasion and physiological states [31]. Specifically, physiological states encompass the interplay between self-efficacy and behavior through physiological arousal when individuals are striving for goals. As stress is a kind of negative emotion, it may have a negative influence on students’ academic self-efficacy. Empirical research has shown that stress negatively predicts academic self-efficacy. For instance, a survey of 197 students revealed that fewer stressors were significant predictors of academic self-efficacy and that academic self-efficacy mediated the effect of stress on academic achievement [57]. In a study that explored the academic self-efficacy of nursing college students, acculturative stress directly affects academic self-efficacy, while e-learning stress indirectly affects students’ academic self-efficacy [58]. An increase in financial pressure on college students results in a decrease in academic self-efficacy [59]. Second, there is a unidirectional prediction of academic self-efficacy on stress [60]. Improving the academic self-efficacy of students promotes their understanding of their abilities and makes them more receptive to negative experiences, so their stress levels are relatively low [61]. Empirical evidence from different age groups supported this finding. For example, improving adolescents’ academic self-efficacy can reduce stress and cultivate learning strategies [62]. For elementary school students, a decrease in self-efficacy can even lead to depression [63]. Moreover, academic self-efficacy regulates the direct impact of academic stress on academic burnout, and higher academic self-efficacy buffers the negative effects of academic stress [43]. During the COVID-19 pandemic, supportive university environments and academic self-efficacy were protective factors against students’ stress and anxiety [64]. Task self-efficacy, a component of academic self-efficacy, has emerged as a significant predictor of students’ academic stress [65]. However, there is little experimental evidence that stress predicts academic self-efficacy among elite college students or vice versa.

### 1.2. The Current Study

Although previous research has extensively discussed the relationship between stress and academic self-efficacy, the longitudinal relationship between stress and academic self-efficacy in elite college students remains unclear. The current study used cross-lagged models to clarify the longitudinal relationship and directionality of the relationship between stress and academic self-efficacy among Chinese students at elite colleges, aiming to improve the psychological health of elite college students. According to the theoretical basis and previous empirical research, we propose the following hypotheses:

**Hypothesis 1:**  *Among Chinese students at elite colleges, stress decreases while academic self-efficacy increases from the junior year to the senior year.*

**Hypothesis 2:**  *Stress is negatively correlated with academic self-efficacy among Chinese students at elite colleges.*

**Hypothesis 3:**  *Stress significantly negatively predicts academic self-efficacy among Chinese students at elite colleges.*

**Hypothesis 4:**  *Academic self-efficacy significantly negatively predicts stress among Chinese students at elite colleges.*

## 2. Materials and Methods

### 2.1. Participants

This study used the data of students from five elite colleges in China and analyzed their information during their junior and senior years [66,67,68,69]. Using stratified, multi-stage and probability proportional sampling to scale methods, the dataset tracked college students from 15 universities in Beijing. As universities included in the 985 Project are prioritized and supported by the Chinese government, aiming to achieve world-class standards, we used their participation in this initiative as the criterion for elite universities. Consequently, we selected student samples from five elite Chinese universities in the dataset, including Peking University, Tsinghua University, Renmin University of China, Beihang University and Beijing Institute of Technology, as the subjects of study. The data were collected at two time points with a one-year interval between them. At Time 1, 919 students participated in the study and completed measures of stress and academic self-efficacy. One year later, at Time 2, 812 students completed the survey. There were no significant differences between students at Time 1 and students who dropped out at Time 2 in terms of gender (t = 0.620, *p* > 0.05), age (t = −1.186, *p* > 0.05), stress (t = −0.262, *p* > 0.05) and academic self-efficacy (t = 0.773, *p* > 0.05), suggesting that the dropouts did not influence the results.

### 2.2. Methods

Stress: The stress of college students was measured using the stress subscale of the Depression Anxiety and Stress Scale 42 (DASS-42) [70]. The subscale consists of 14 items, and a 3-point Likert scale ranging from 0 to 3 was used for each item. The total score for the 14 items ranges from 0 to 42, with higher scores indicating higher levels of stress. Scores within the range of 0–14 correspond to normal stress levels, while scores of 15–18 indicate mild stress, scores of 19–25 indicate moderate stress, scores of 26–33 indicate severe stress and scores above 34 indicate extremely severe stress. The alpha coefficients for stress at Time 1 and Time 2 were 0.8975 and 0.9081, respectively.

Academic self-efficacy: The academic self-efficacy of college students was evaluated by the academic self-efficacy subscale of the Patterns of Adaptive Learning Scales (PALS), which includes five items [71]. Each item was rated on a 5-point Likert scale ranging from 1 (Not at all true of me) to 5 (Completely true of me). The total score for the 5 items ranges from 5 to 25, with higher scores indicating higher levels of academic self-efficacy. The alpha coefficients for academic self-efficacy at Time 1 and Time 2 were 0.8694 and 0.8536, respectively.

### 2.3. Data Analysis

**Figure 1:**
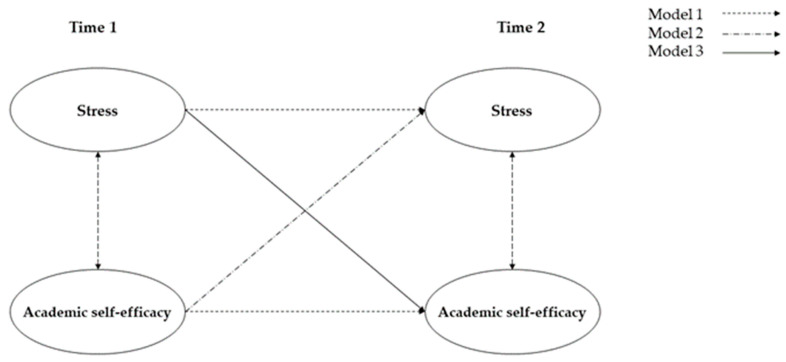
A cross-lagged model depicting the relationship between stress and academic self-efficacy across two distinct time periods. Note: Model 1 is the autoregressive model. Model 2 included autoregression and the predictive effects of academic self-efficacy on stress. Model 3 included autoregression and the predictive effects of stress on academic self-efficacy. Model 4 incorporated all the autoregressive and cross-lagged paths of Model 1, Model 2 and Model 3. Model 5 extended upon Model 4 by integrating demographic variables. In the same year, two variables are correlated.

This study employed Stata 15.0 and Mplus 7.4 for data analysis and processing. First, descriptive and correlation analyses were conducted for stress and academic self-efficacy. Subsequently, we constructed five models (see Figure 1) using Mplus 7.4 to examine the following relationships between stress and academic self-efficacy: (1) stress significantly negatively predicts academic self-efficacy, and (2) academic self-efficacy significantly negatively predicts stress. The model fit was evaluated using various fit indices [72]. Generally, a well-fitting model is indicated by (1) the comparative fit index (CFI) > 0.90 (superior fit ≥ 0.95), (2) the Tucker–Lewis index (TLI) > 0.90 (superior fit ≥ 0.95), (3) the root mean square error of approximation (RMSEA) < 0.10 (superior fit ≤ 0.06) and (4) the standardized root mean square residual (SRMR) < 0.10 [73].

## 3. Results

### 3.1. Descriptive Statistics and Correlation Analysis between Stress and Academic Self-Efficacy

Table 1 presents the descriptive statistics and correlation analysis of stress and academic self-efficacy. The results showed that there was a marginal decrease in the mean values of stress from Time 1 to Time 2, as well as a slight increase in academic self-efficacy, suggesting a modest change over time. These two variables were significantly and negatively correlated at Time 1 (r = −0.155, *p* < 0.05) and Time 2 (r = −0.241, *p* < 0.05). In addition, a significant negative correlation was found between academic self-efficacy at Time 1 and stress at Time 2 (r = −0.137, *p* < 0.05) as well as between stress at Time 1 and academic self-efficacy at Time 2 (r = −0.177, *p* < 0.05).

### 3.2. Cross-Lagged Analysis between Stress and Academic Self-Efficacy

Based on the analysis above, we used a cross-lagged model to examine the predictive relationship between stress and academic self-efficacy. Table 2 presents the fit indices for the five models, while Table 3 displays the autoregressive path coefficients and cross-lagged path coefficients.

Model 1 was the autoregressive model and fit well (χ^2^ = 198.462, CFI = 0.980, RMSEA = 0.057, SRMR = 0.042). It was employed to evaluate the stability of both stress and academic self-efficacy. This model encompassed autoregressive paths of stress and academic self-efficacy. According to Table 3, the stability paths of stress (β = 0.675, *p* < 0.05) and academic self-efficacy (β = 0.587, *p* < 0.05) were both significant from Time 1 to Time 2.

Model 2 exhibited a quite acceptable fit (χ^2^ = 195.590, CFI = 0.980, RMSEA = 0.057, SRMR = 0.038). It was used to examine whether academic self-efficacy at Time 1 could predict stress at Time 2. This model included autoregressive paths of stress and academic self-efficacy, as well as a cross-lagged path from academic self-efficacy at Time 1 to stress at Time 2. The analysis presented in Table 3 showed that stress (β = 0.666, *p* < 0.05) and academic self-efficacy (β = 0.593, *p* < 0.05) remained stable over time. Moreover, academic self-efficacy at Time 1 did not significantly predict stress at Time 2 (β = −0.052, *p* > 0.05).

Model 3 was employed to examine whether stress at Time 1 could predict academic self-efficacy at Time 2 (χ^2^ = 187.542, CFI = 0.981, RMSEA = 0.055, SRMR = 0.028). This model incorporated autoregressive paths of stress and academic self-efficacy, along with a cross-lagged path from stress at Time 1 to academic self-efficacy at Time 2. As shown in Table 3, the analysis revealed the stability of stress (β = 0.684, *p* < 0.05) and academic self-efficacy (β = 0.568, *p* < 0.05) from Time 1 to Time 2. Furthermore, the cross-path coefficient from stress at Time 1 to academic self-efficacy at Time 2 were statistically significant (β = −0.112, *p* < 0.05).

Model 4 was used to examine the mutual influence between stress and academic self-efficacy. All the autoregressive paths and cross-lagged paths from Model 1, Model 2 and Model 3 were included. The results of the chi-square difference test indicated that Model 4 demonstrated the best fit (χ^2^ = 184.883, CFI = 0.982, RMSEA = 0.056, SRMR = 0.025) compared to Model 1, Model 2 and Model 3. As illustrated in Table 3, both stress (β = 0.675, *p* < 0.05) and academic self-efficacy (β = 0.574, *p* < 0.05) remained stable from Time 1 to Time 2. Moreover, stress at Time 1 significantly predicted academic self-efficacy at Time 2 (β = −0.111, *p* < 0.05).

Model 5 extended upon Model 4 by integrating demographic variables such as age, gender, race, character and family social status to assess their impact on the bidirectional connection between stress and academic self-efficacy. This model fit well (χ^2^ = 253.527, CFI = 0.978, RMSEA = 0.045, SRMR = 0.021). Academic self-efficacy at Time 1 did not significantly predict stress at Time 2 (β = −0.047, *p* > 0.05), but stress at Time 1 significantly predicted academic self-efficacy at Time 2 (β = −0.102, *p* < 0.05). These findings suggest that the unidirectional cross-lagged effect between stress and academic self-efficacy remained stable even after incorporating demographic variables.

In summary, Model 1 elucidated the stability of academic self-efficacy and stress from Time 1 to Time 2. Model 2 revealed that academic self-efficacy at Time 1 did not significantly predict stress at Time 2. Model 3 indicated that stress at Time 1 significantly predicted academic self-efficacy at Time 2. Model 4 and Model 5 further confirmed that stress unidirectionally predicted academic self-efficacy over time. However, academic self-efficacy did not significantly predict stress.

## 4. Discussion

The main contribution of this paper was to reveal the unidirectional prediction of stress on academic self-efficacy among Chinese students at elite colleges.

First, descriptive statistics showed that there was a slight decrease in stress from the junior year to the senior year, which supported hypothesis 1. Previous research has shown that students‘ stress is not stable during the four years of college [9,10]. The development of stress among college students followed different paths, and the stress of most students showed a decreasing trend from junior to senior years, which is consistent with the results of this study [11]. A survey of 421 medical students of all academic years also revealed that stress significantly decreased as students progressed from the junior year to the senior year [2]. This might be attributed to the following reasons. First, compared to junior students, senior students may acquire more learning skills and achieve success in their senior year, so the confidence brought about by success will reduce stress [74]. Second, students may improve their social skills and become adept at interpersonal relationships [75]. Thus, they can effectively resolve disagreements when they confront social conflicts [76]. Third, although the income of elite college students may not necessarily be high, they can still have an advantage in the job market due to the reputation of elite colleges and their outstanding abilities. Therefore, for elite university students in their senior year, employment pressure may not be significant. Additionally, it is noteworthy that existing research has demonstrated fluctuations in various psychological states among college students during their university years [77]. The data used in this study were obtained from surveys conducted during the third and fourth years of college. Given that undergraduate education in China typically spans four years, with most of the coursework concentrated in the first three years and the fourth year allocated for job searching and graduation project design, many students experience reduced academic pressure during their fourth year. This reduction in stress may be considered a normal phenomenon in the Chinese context. However, considering the increasing challenges in recent years for Chinese college graduates to secure employment, it is possible that, in updated data, stress levels during the fourth year may not necessarily decrease. This represents a crucial issue for further investigation in the future.

Second, Chinese students at elite colleges experienced a marginal increase in academic self-efficacy, which verified hypothesis 1. The results are consistent with previous research showing that the academic self-efficacy of college students experienced a downward trend from the first year to the third year of college but rose slightly in the senior year [37]. First, academic self-efficacy is positively correlated with academic achievement [28]. After one year of study, students may achieve academic success in the senior year, and their self-evaluations tend to be more positive, thus improving their academic self-efficacy. Second, academic self-efficacy is positively correlated with academic engagement [78]. Chinese college students have basically completed the courses required for graduation from their freshman to junior year. Therefore, in their senior year, they can invest more time and energy in the learning content they are interested in. As a result, their academic self-efficacy may slightly improve.

Third, stress and academic self-efficacy were negatively correlated among Chinese students at elite colleges, which has been made evident in previous studies underlining the correlation between stress and academic self-efficacy [29,42]. The results verified hypothesis 2 proposed in our study. Immigrant and ethnic minority students, like elite college students, represent a distinct demographic group. A study on immigrants and minority college students in the United States has reached the same conclusion that stress and academic self-efficacy are negatively correlated [27]. On the one hand, students under high pressure may have low academic self-efficacy and lack sufficient confidence in their learning and problem-solving abilities [42]. Conversely, students facing less pressure tend to have a more positive subjective evaluation of themselves [56]. On the other hand, higher levels of academic self-efficacy may also signify increased confidence and greater psychological resilience in viewing future academic tasks as challenges rather than threats, thus exhibiting a negative correlation with stress [42]. In other words, students who maintain a positive outlook on future challenges tend to experience lower levels of stress. High academic self-efficacy is also associated with superior academic performance, increasing the likelihood of success [79,80]. Therefore, successful experiences amplify self-assurance and lower stress among students.

Fourth, stress significantly negatively predicted academic self-efficacy among Chinese students at elite colleges, which confirmed hypothesis 3. Previous research has shown that an increase in stress leads to a decrease in academic self-efficacy among college students, which is consistent with our findings [59]. According to Bandura’s theory, four factors influence self-efficacy including practical experiences, vicarious experiences, verbal persuasion and physiological states. Therefore, stress, a form of negative emotion, may significantly influence academic self-efficacy [31]. Chinese elite college students, in particular, confront not only intense academic competition but also high expectations from parents and teachers, so they may be under much pressure, which has a negative influence on their academic performance and psychological health. In this way, their assessment of personal abilities tends to be underestimated, and academic self-efficacy tends to be weak.

Fifth, academic self-efficacy did not significantly predict stress, which is contrary to hypothesis 4. These findings were not consistent with prior findings, and the direction of the impact between stress and academic self-efficacy needs to be further verified [60]. This study revealed that only stress significantly predicted a negative outcome for academic self-efficacy, but the opposite effect was not significant. One possible reason for the difference in results could be the sample selection. Students who are able to attend elite universities in China are selected through highly competitive exams, so they already have strong self-belief before entering university. This belief enables students from elite universities to better cope with stress. Therefore, changes in academic self-efficacy may not have a significant effect on stress. In addition, the excellence of elite college students is reflected not only in academic performance but also in emotional intelligence and interpersonal communication [39]. A survey of 1315 college students found that regulatory emotional self-efficacy was negatively correlated with academic stress. Therefore, we assumed that students from elite colleges may alleviate stress through emotional regulation or communication with others such as teachers or friends. In this way, the impact of academic self-efficacy on stress is not significant.

## 5. Limitations

First, despite utilizing scales with high reliability and validity to measure stress and academic self-efficacy, the data collected in this study relied solely on self-reported measures. However, self-reported measures tend to be influenced by students’ emotions and the surrounding environment, which may affect the results. Therefore, future research should adopt other measurement methods.

Second, the participants in this study were college students from five elite colleges in China. Stress and academic self-efficacy may differ among college students from other types of universities in China, other countries or from different cultural backgrounds.

Third, this study only used data from two time points. Further investigations should record changes in stress and academic self-efficacy among elite college students over a longer period of time or at more time points.

## 6. Implications for Educational Practice and Conclusions

### 6.1. Implications for Educational Practice

Stress and academic self-efficacy are important predictive factors for the psychological health of college students [81,82]. The current study investigated the longitudinal relationship between stress and academic self-efficacy among Chinese students at elite colleges. This study thereby complemented the literature while shedding invaluable light on strategies to alleviate students’ stress.

First, this study’ findings indicated that stress and academic self-efficacy were negatively correlated among Chinese elite college students. Therefore, improving students’ academic self-efficacy is beneficial for their stress alleviation. Previous research has shown that practical experiences, vicarious experiences, verbal persuasion and physiological states influence students’ academic self-efficacy [31]. By presenting positive and diligent role models in learning and delivering compelling guidance, we can effectively improve students’ academic self-efficacy. Furthermore, self-esteem is positively correlated with academic self-efficacy and positively predicts academic self-efficacy among Chinese college students. We can cultivate students’ self-esteem to improve their academic self-efficacy [83]. In addition, students should actively engage in interpersonal communication and develop comprehensive university plans to enhance their mental well-being and academic self-efficacy [65].

Second, we can promote students’ mental health through life support and the establishment of a positive mental health atmosphere. For example, the guiding role of mentors should be harnessed throughout the academic journey, and comprehensive attention should be given to diverse aspects of students’ academic growth, as well as offering timely assistance and guidance when students encounter academic or life challenges [84]. Furthermore, there is a negative correlation between family cohesion, interpersonal relationships and stress [85,86]. Therefore, the support and assistance of family and classmates play an important role in the mental health of students. Additionally, courses related to stress relief and academic guidance should be introduced, knowledge on stress management and academic planning should be disseminated to students and the correct methods for stress relief should be guided to help students enhance their crisis response, life planning and stress relief abilities through understanding scientific concepts and methods [87]. Mindfulness training and outdoor activities are beneficial for relieving student stress and adapting to the role transformation of college students [3,88].

Third, the application of artificial intelligence technology in stress intervention should be enhanced. We can establish an imperceptible and user-friendly AI psychological intervention system. The system should be effective in providing psychological support to students who may be experiencing stress or other mental health challenges. In addition, AI can provide early warnings to students and educators about potential mental health issues through analyzing students’ academic performance and social interactions. In this way, mental health issues can be addressed before they become worse. Moreover, artificial intelligence should provide a range of services from identifying one’s stress to providing professional counseling. It can help to address mental health issues by providing students with information and resources to better understand their own mental state and the importance of mental health. This can contribute to creating a more positive and informed mental health culture within the educational community. Furthermore, the AI psychological intervention system should be adaptable and personalized to each student’s needs. It should take into account individual differences, cultural sensitivities and personal preferences to ensure that the support provided is both relevant and effective. In the future, interventions for student stress should pay more attention to a positive mental health atmosphere in the educational environment and the application of technology.

### 6.2. Conclusions

First, students experienced a slight reduction in stress and a marginal increase in academic self-efficacy from the junior year to the senior year. Second, there was a significant negative correlation between stress and academic self-efficacy among Chinese elite college students. Third, stress exhibited a unidirectional negative prediction over time on academic self-efficacy among Chinese students at elite colleges. In the future, higher education institutions should pay more attention to cultivating students’ psychological health and academic self-efficacy, creating a healthy atmosphere and promoting their future development.

## Figures and Tables

**Table 1:** Descriptive statistics and correlation coefficients of stress and academic self-efficacy.

Variables	1	2	3	4
1. Stress (T1)	1			
2. Stress (T2)	0.622 *	1		
3. Academic self-efficacy (T1)	−0.155 *	−0.137 *	1	
4. Academic self-efficacy (T2)	−0.177 *	−0.241 *	0.529 *	1
Mean	12.320	11.603	18.751	18.849
Standard deviation	7.982	8.041	4.254	3.907

Note: T1, measurement time 1; T2, measurement time 2. * *p* < 0.05.

**Table 2:** Model fit indices of stress and academic self-efficacy.

Model	χ^2^	df	RMSEA (90% CI)	SRMR	CFI	TLI	Comparison	∆χ^2^	*p*
1	198.462	50	0.057 (0.049–0.065)	0.042	0.980	0.974			
2	195.590	49	0.057 (0.049–0.066)	0.038	0.980	0.973	Model 1–Model 2	2.872	<0.05
3	187.542	49	0.055 (0.047–0.064)	0.028	0.981	0.975	Model 1–Model 3	10.920	<0.05
4	184.883	48	0.056 (0.047–0.064)	0.025	0.982	0.975	Model 1–Model 4	13.579	<0.05
5	253.527	88	0.045 (0.039–0.052)	0.021	0.978	0.968	Model 1–Model 5	−55.065	<0.05

Note: χ^2^ = chi-square; df = degree of freedom; RMSEA = root mean square error of approximation; SRMR = standardized root mean square residual; CFI = comparative fit index; TLI = Tucker–Lewis index.

**Table 3:** Standardized autoregressive path and cross-lagged path coefficients.

Model	Autoregressive Path	β	Cross-Lagged Path	β
1	Stress (T1) → Stress (T2)	0.675 *		
	Academic self-efficacy (T1) → Academic self-efficacy (T2)	0.587 *		
2	Stress (T1) → Stress (T2)	0.666 *	Academic self-efficacy (T1) → Stress (T2)	−0.052
	Academic self-efficacy (T1) → Academic self-efficacy (T2)	0.593 *		
3	Stress (T1) → Stress (T2)	0.684 *	Stress (T1) → Academic self-efficacy (T2)	−0.112 *
	Academic self-efficacy (T1) → Academic self-efficacy (T2)	0.568 *		
4	Stress (T1) → Stress (T2)	0.675 *	Stress (T1) → Academic self-efficacy (T2)	−0.111 *
	Academic self-efficacy (T1) → Academic self-efficacy (T2)	0.574 *	Academic self-efficacy (T1) → Stress (T2)	−0.050
5	Stress (T1) → Stress (T2)	0.671 *	Stress (T1) → Academic self-efficacy (T2)	−0.102 *
	Academic self-efficacy (T1) → Academic self-efficacy (T2)	0.574 *	Academic self-efficacy (T1) → Stress (T2)	−0.047

Note: β = standardized coefficient. * *p* < 0.05.

## Data Availability

The data that support the findings of this study are available from the corresponding author upon reasonable request.
